# Prolonged urinary retention in COVID-19 survivors: observational study

**DOI:** 10.1590/1806-9282.20240845

**Published:** 2024-11-11

**Authors:** Natália Dalsenter Avilez, Lucas Mira Gon, Vinicius Camargo Achermann, Cassio Luis Zanettini Riccetto

**Affiliations:** 1 Universidade Estadual de Campinas – Campinas (SP), Brazil.

**Keywords:** COVID-19, SARS-CoV-2, Coronavirus, Voiding dysfunction, Lower urinary tract symptoms, Urinary retention

## Abstract

**OBJECTIVE:**

Initially diagnosed as a respiratory disease, SARS-CoV-2 revealed numerous extrapulmonary implications. The aim of this study was to investigate prolonged urinary retention in survivors post-COVID-19 infection that led to hospitalization.

**METHODS:**

A retrospective cohort analysis included male and female patients hospitalized during the COVID-19 pandemic in a reference center hospital. Exclusions were patients with a history of lower urinary tract surgeries or symptoms, including urinary incontinence, those using medication affecting detrusor contractile activity, and those with established neurological diseases. Clinical, laboratory, and radiographic data were obtained from medical records and analyzed using chi-square, Fisher's exact, Mann-Whitney, Kruskal-Wallis, and Dunn tests.

**RESULTS:**

The study included 834 patients, with 471 (56.5%) male and 363 (43.5%) female. Of these, 300 patients used a urinary catheter, and 12.6% were unable to remove it due to sustained urinary retention. Orotracheal intubation, thrombocytopenia, urinary tract infections, and higher Sequential Organ Failure Assessment scores were associated with urinary retention. Correlation analysis showed that the highest percentage of pulmonary involvement on computed tomography was related to longer catheterization time and failed attempts to remove the catheter, affecting men and women equally.

**CONCLUSIONS:**

Urinary tract involvement in COVID-19 infection is increasingly evident. The correlation between COVID-19 severity and failure to remove the urinary catheter in a similar percentage of men and women reinforces the hypothesis that sex-independent urothelial injury and bladder dysfunction might be caused by COVID-19.

## INTRODUCTION

In December 2019, the SARS-CoV-2 infection emerged in China and spread around the world^1^. While initially identified for its respiratory manifestations akin to viral pneumonia, subsequent investigations have revealed numerous extrapulmonary implications. The route through which the virus enters the host cell can help us understand its impact on different organs. This process relies on the angiotensin-converting enzyme receptor (ACE2)^1,2^, whose expression is high in the lungs, intestines, and kidneys, but also significant in urothelial cells^3^. This makes the lower urinary tract a potential target for SARS-CoV-2 infection. The virus’ entry into the host cell triggers an inflammatory cascade, also called a "cytokine storm," which leads to conditions like cystitis^4^. Pro-inflammatory mediators, including the cytokines themselves, might cause damage to the bladder mucosa and activate afferent nerves, which might result in lower urinary tract symptoms (LUTS) and compromise bladder function, similarly as demonstrated in other bladder diseases^5^.

Some studies reported urinary tract involvement in patients post-COVID-19 pandemic. Similarly, there were descriptions of increased urine frequency^6^ and prolonged expression of pro-inflammatory cytokines in the urine post-SARS-CoV-2 infection^7^. While prior research predominantly explored ­voiding symptoms in men, potentially attributed to SARS-CoV-2 infection impact on the prostate, a comprehensive evaluation considering both sexes was warranted to discern the virus’ effect on the bladder. This study aimed to focus on the risk factors for prolonged urinary retention after SARS-CoV-2 infection.

## METHODS

### Data collection and definition of some indicators

We performed a retrospective cohort analysis including survivors, male and female patients, hospitalized with COVID-19 between April 2020 and July 2021 in our institution. Clinical, laboratory, and radiographic data were obtained from medical records and analyzed to identify risk factors for the development of lower urinary tract dysfunction after SARS-CoV-2 infection.

The inclusion criteria were men and women over 18 years of age admitted with a diagnosis of SARS-CoV-2 infection confirmed by RT-PCR. There were excluded patients with a history of lower urinary tract surgeries, patients with LUTS, women with pelvic organ prolapses, patients using medication with a potential effect on detrusor contractile activity, such as antidepressants, anticholinergics, and muscle relaxants, and patients with neurological diseases.

Data such as age, sex, comorbidities, length of stay, length of stay in the intensive care unit (ICU), mechanical ventilatory support, and its parameters were compiled. Clinical and biomarker assessments were used to calculate the Sequential Organ Failure Assessment (SOFA) score. Intubated patients were categorized according to SOFA score. Pulmonary involvement was ­examined through the percentage and severity of lung involvement on thorax computed tomography (CT). Additionally, the presence or absence of urinary tract infections (UTI) was scrutinized, discerning ­etiological agents through culture identification. Only complete cases, i.e., individuals without missing data for the ­variables of interest, were included in the analysis. This approach was crucial to ensure the integrity and accuracy of our investigation.

The study faced some potential biases. There could be selection bias due to its focus on hospitalized patients, potentially neglecting milder cases. Reporting bias could skew outcomes if selective reporting occurred. Excluding deceased patients before catheter removal could also introduce sampling bias. Varied catheterization durations could lead to duration bias, influencing outcomes. To avoid such biases, the study applied various strategies such as research into diversified data sources, ensured stringent data collection protocols, and implemented strict exclusion criteria to enhance accuracy and minimize potential biases.

### Statistical analysis

The data were analyzed with the Statistical Analysis System (SAS System) for Windows, version 9.4 (SAS Institute Inc., Cary, NC, USA). The statistical descriptive analysis was presented as frequency tables for categorical variables and measures of position and dispersion for continuous variables. To compare proportions, we used the chi-square test and Fisher's exact test when necessary. Continuous measures between two groups were compared using the Mann-Whitney test, and Kruskal-Wallis was used for comparisons among three or more groups, followed by Dunn's test for locating differences, when necessary. The Spearman's linear correlation coefficient assessed the relationship between numerical variables. The adopted ­significance level for the statistical tests was 5%.

## RESULTS

Data presentation is in accordance with the STROBE Statement for observational studies. From an initial cohort of 1,329 COVID-19 hospitalized patients, 834 medical records met the study's inclusion criteria. The exclusion process accounted for specific cases: 273 patients who died prior to catheter removal, 77 with pre-existing neurological conditions, 65 who presented prior LUTS, 45 who were using medications that could impact bladder detrusor function, and 35 who comprised other minor confounders.

The descriptive analysis is summarized in Table 1. Of the total patients, 471 (56.5%) were male and 363 (43.5%) were female. The mean age was 53.9 years and 67.9% had comorbidities, including 29.5% with diabetes mellitus. Although ­hospitalized, almost 80% of the participants did not require intubation and were considered moderate cases. Cases requiring intubation were considered severe and were also evaluated for the pO2/FiO2 ratio and classified according to the SOFA score at the time of admission, so 18.4% had a score of 0–6 and 1.7% had a score of 7–18. In addition, all patients were evaluated for the percentage of pulmonary involvement on chest tomography, with the following results: 20.4% had less than 25% of the lungs affected; 45.7% between 25 and 50%; 27.4% between 50 and 75%; and 6.6% more than 75% of the lungs affected.

**Table 1:** Descriptive analysis of general data.

Variables		Mean	Median	Results			
				SD	Mx	Mn	n
Age		53.9	55	14.76	95	18	810
Number of days in ICU		14.51	11	12.49	73	0	200
Days with indwelling vesical catheter		6.22	0	11.77	85	0	730
			(n)			(%)	
Sex							
	Female		363			43.5	
	Male		417			56.5	
Diabetes			246			29.5	
Hematuria			12			1.4	
UTI—confirmed by exam			95			11.4	
Intubated			161			20.3	
Catheterization			300			39.7	
Removal of the indwelling vesical catheter							
	Removed		262			35.6	
	Not removed		38			5	
Percentage of pulmonary involvement on thorax computed tomography (%)							
	<25		87			20.4	
	25–50		195			45.7	
	50–75		117			27.4	
	>75		28			6.6	
pO_2_/FiO_2_ ratio							
	<100		18			11.3	
	100–200		84			52.5	
	200–300		22			13.8	
	300–400		14			8.8	
	≥400		22			13.8	
SOFA score (just intubated)							
	0–6		146			18.4	
	7–18		14			1.7	

SD: standard deviation; ICU: intensive care unit; UTI: urinary tract infections; SOFA: Sequential Organ Failure Assessment; pO2: partial pressure of alveolar oxygen; FiO2: fraction of inspired oxygen..

Out of the total number of patients, 300 used a urinary ­catheter, and 12.6% of them evolved with sustained urinary ­retention even after the recovery of respiratory conditions. Table 2 presents the comparison between patients who had success or not in catheter removal. Orotracheal intubation (p=0.0020), low platelets count (p=0.0065), UTI (p=0.0290), and higher SOFA (p=0.0009) scores were all associated with urinary retention, and age, sex, and the presence of diabetes were not associated.

**Table 2:** Analysis and comparisons between success or not to remove the urinary catheter.

Variables		Removed				Not removed				p-value
		Mean	SD	Range	n	Mean	SD	Range	n	
Age		55.38	13.82	20–88	249	57.06	15.16	19–82	36	0.3377
Number of days in hospital		25.52	19.51	2–185	261	30.74	18.02	4–73	38	0.057
Number of days in ICU		16.25	12.01	0–72	187	19.46	16.03	0–73	35	0.0401
			(n)	(%)			(n)	(%)		p-value
Sex			114	43.5			16	42.1		0.8701
	Female		148	56.5			22	57.9		
	Male									
Diabetes										
	No		178	67.9			22	57.9		0.2197
	Yes		84	32.1			16	42.1		
Hematuria										
	No		254	96.9			34	89.5		0.0513
	Yes		8	3.1			4	10.5		
Intubated										
	No		110	45.6			6	17.6		0.002
	Yes		131	54.4			28	82.4		
UTI—confirmed by exam										
	No		193	74.8			22	57.9		0.029
	Yes		65	25.2			16	42.1		

SD: standard deviation; ICU: intensive care unit; UTI: urinary tract infections.

Statistically significant values are indicated in bold.

**Figure 1:**
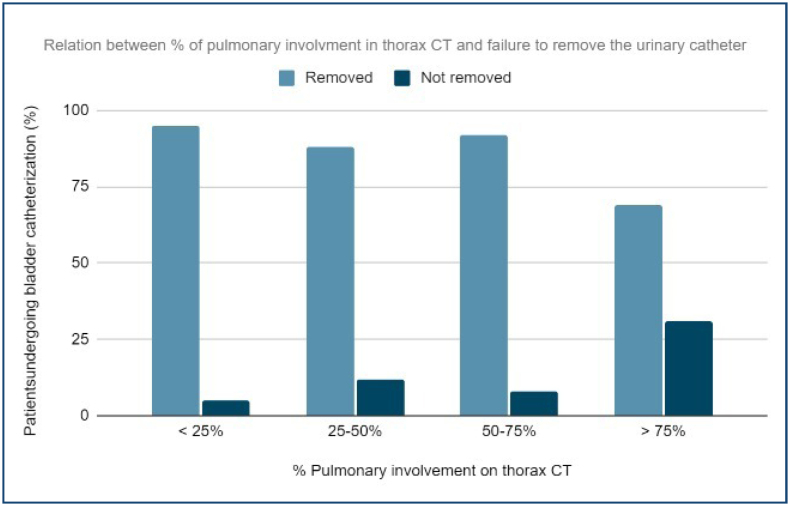
Relation between % of pulmonary involvment in computed tomography and failure to remove the urinary catheter.

Voiding dysfunction is also significantly associated with the severity of pulmonary involvement (p<0.0001), and the correlation analysis showed that the higher percentage of pulmonary involvement on CT was related to longer catheterization time and failed attempts to remove the urinary catheter (Figure 1).

UTI was also correlated with SOFA scores above 6. This group of more severe cases also had a longer catheter time, which is known to be a risk factor itself. Macroscopic ­hematuria was present in 1.4% of the patients, and its incidence was higher in patients who had UTIs and SOFA scores above 6.

## DISCUSSION

Some studies analyzed the effect of SARS-CoV-2 infection on the urinary tract. Most of them focused on the male urinary tract and were conducted in urological centers, which concluded that the infection led to an increase in the incidence of ­previous LUTS. To the best of our knowledge, the present study is the first to focus on both male and female patients, with no previous urinary symptoms, and was carried out in a large center that was a reference for COVID-19 during the pandemic.

Almost 40% of the patients underwent urinary catheterization during hospitalization, and its duration was correlated with the severity of SARS-CoV-2 infection. The failure to remove the urinary catheter due to urinary retention was observed in 5% of all patients, which accounted for 12.6% of those who required a urinary catheter. Notably, a similar proportion of males (12.9%) and females (12.3%) exhibited urinary retention upon catheter removal, persisting even after recovery from SARS-CoV-2 pulmonary infection. These numbers were unexpected since urinary retention is a condition more frequently found in men than in women, and support the hypothesis that SARS-CoV-2 infection can cause bladder dysfunction. This opposes the hypothesis formulated by Haghpanah et al.^11^, which suggested that voiding dysfunction after COVID-19 ­infection occurred due to exacerbation of bladder outlet obstruction related to benign prostatic hyperplasia, since, in this case, we would not expect such an effect in the female population.

The incidence of urinary retention was observed more ­frequently in patients with higher percentages of lung involvement on CT. The SOFA score was also significantly correlated with a higher risk of urinary retention, and both findings indicate possible direct effects of SARS-CoV-2 on the urothelium, which is related to the severity of the viral lung infection but that lasts longer than the acute pulmonary condition, as reported by Lamb et al.^9^.

Some studies report additional types of urinary tract involvement in patients with COVID-19^6-8^. A systematic review of 16 studies and 557 patients infected with COVID-19 found that 43 patients had LUTS and 7 had deterioration of pre-existing symptoms. Overactive bladder was the most frequent ­problem, followed by hematuria, urinary retention, microhematuria, and leukocyturia^8^. Another study found that of 57 patients ­hospitalized in specialized wards for COVID-19, 7 presented increased urinary frequency as a complaint at admission^7^.

Can et al. evaluated the IPSS of 94 participants hospitalized due to SARS-CoV-2 infection, who were divided into two groups according to age. Patients over the age of 50 had an IPSS of 5.1±4.1 before infection, which increased to 9±6.4 during the disease12. A smaller study, which included younger patients with mean ages of 32 and 39, respectively for female and male patients, found that LUTS were more prevalent during the acute phase of COVID-196. In another study, which focused on a cohort of pediatric urology patients, it was observed that incomplete bladder emptying and urinary retention were supported by an increased bladder compliance and high volumes of post-voiding residual^13^. Our cohort ranged in age from 18 to 95 years, with a median of 55 years old, and found no relationship between age and urinary retention, but also hematuria and UTIs. Other variables, such as sex and concomitance of conditions such as diabetes, which could be related to voiding dysfunctions, were not associated with LUTS.

Despite not yet completely elucidated, several possibilities are currently considered for the mechanism of urothelial involvement. The pathophysiology of SARS-CoV-2 involves binding the virus spike protein to ACE2 receptors on pneumocytes. Although lung cells with ACE2 expression are the main target cells of the infection, a study identified significant ACE2 expression in kidney proximal tubule cells with the proportion of ACE2-positive cells at approximately 4%. Furthermore, the proportion of ACE2-positive cells in the bladder urothelial cells was 2.4%^3^. Therefore, kidney and ­bladder were ­considered high risk, especially in viremia sets. The ­recruitment of immune cells, either by direct viral infection of the endothelium or immune-mediated, can result in widespread endothelial dysfunction associated with apoptosis. This finding may explain the urinary symptoms observed in COVID-19 patients. Another hypothesis suggests a direct insult to the bladder or urothelium leading to viral cystitis^8^. As in other infectious cystitis, mast cells are activated by numerous mediators, such as the cytokine-like stem cell factor, that are released by the damaged urothelium. Once activated, mast cells undergo degranulation and release vasoactive, inflammatory, and nociceptive mediators such as histamine, cytokines, leukotrienes, prostaglandins, and nitric oxide. Those mediators stimulate protease-activated receptors in the tissue, leading to widespread inflammation and neuronal hyperexcitability, which cause more urothelial inflammation and urinary symptoms^5^. Thus, urinary symptoms occur in severe disease, in the long-term, or in post-acute COVID-19 syndrome (PACS)^9^. Increased pro-inflammatory cytokines were detected in COVID-19 patients with severe urinary symptoms, suggesting a relation between COVID-19 inflammation and bladder dysfunction^10^.

It is currently unclear whether LUTS in the COVID-19 setting is reversible in the long term. Prospective multicenter ­studies with long-term follow-ups are needed to clarify these issues. Future studies, including detailed urodynamic studies and cystoscopy assessments, may help to address the pathophysiology.

## CONCLUSION

COVID-19 infection can cause persistent urinary retention in men and women in similar proportions, suggesting that such voiding dysfunctions occur due to injury to the bladder urothelium. Moreover, there is a significant correlation between respiratory ­severity and urinary tract dysfunction, manifested by urinary retention.

## ETHICAL STANDARDS

The authors certify that the study was performed under the ­ethical standards as laid down in the 1964 Declaration of Helsinki and its later amendments or comparable ethical standards. Certificate of Presentation of Ethical Review by Unicamp Research Ethics Committee number: 51408321.1.0000.5404. Approval at 23/08/2022.
